# Solid Pseudopapillary Neoplasm of the Pancreas

**DOI:** 10.5334/jbr-btr.1157

**Published:** 2016-09-07

**Authors:** Ieva Garkalne, Carola Brussaard

**Affiliations:** 1 UZ Brussel, BE

**Keywords:** solid pseudopapillary neoplasm of pancreas, gastric GIST, rare pancreatic tumour, abdominal MRI, long standing abdominal pain

A 31-year-old Caucasian woman suffered from severe recurrent pain in the upper abdomen, nausea and epigastric pain crisis for a period of about 10 years. On her own initiative, she visited the emergency department. On physical examination pain and tenderness on right paralumbar area was noted. Blood analyses failed to show any significant abnormality. She was scheduled for computed tomography (CT) to rule out nephrolithiasis or a lumbar disc hernia. Abdominopelvic CT revealed a heterogeneous mass of 10 x 8.5 cm in the left upper quadrant. The mass showed continuity toward the tail of the pancreas (Figure 1, black arrowheads) and probably also to the stomach wall (Figure 1, white arrowheads), but no sign of invasion was noted. The mass was well circumscribed, equally cystic and solid and showed no calcification. After intravenous contrast the surrounding capsule and the solid components showed enhancement (Figure 1, star). Neither abnormal locoregional lymph nodes nor distant metastases were demonstrated.

**Figure 1:**
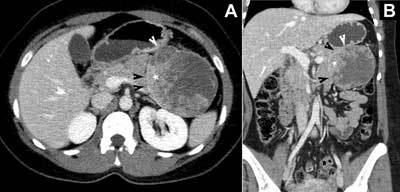
Contrast enhanced abdominal CT. Axial image **(A)** and coronal image **(B)** showing a large heterogeneous mass with contrast enhancing solid components (star). The mass showing continuity toward the tail of the pancreas (black arrowheads) and probably also to the stomach wall (white arrowheads).

A complementary magnetic resonance imaging (MRI) showed a large, mostly solid, well-demarcated lobulated mass, heterogeneous on T1- and T2-weighted images. An intralesional haemorrhage was noted as hyperintense areas on T1-weighted images with fat saturation (Figure 2A, black star). Dynamic contrast confirmed slow enhancement of the solid regions (Figure 2B, white star) and surrounding capsule (Figure 2B, white arrowheads). Diffusion-weighted imaging (Figure 3A) and apparent diffusion coefficient map (Figure 3B) showed focal diffusion restriction in the solid components (Figure 3, white arrowheads).

**Figure 2:**
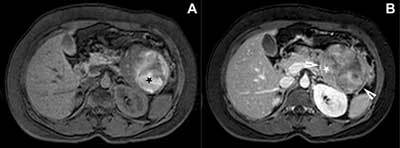
Abdominal MRI. Axial T1-weighted image with fat saturation **(A)** showing hyperintense areas due to intralesional haemorrhage. Axial contrast enhanced T1-weighted image with fat saturation **(B)** displaying slow enhancement of the solid regions (white star) and surrounding capsule (white arrowheads).

**Figure 3:**
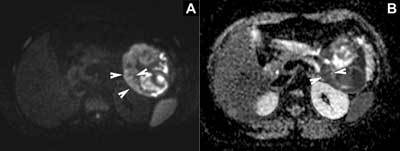
Abdominal MRI. Axial diffusion-weighted imaging **(A)** and apparent diffusion coefficient map **(B)** showing focal diffusion restriction in the solid components (white arrowheads).

Under the provisional diagnosis of a gastric gastrointestinal stromal tumor (GIST), laparotomy was performed. At surgery the pancreatic origin of the tumour was established and distal pancreatectomy with splenectomy was carried out.

The diagnosis of solid pseudopapillary neoplasm (SPN) of the pancreas was made by pathological examination postoperatively. The patient didn’t receive adjuvant treatment. Follow-up abdominal CT six months after surgery showed neither recurrence nor distant metastases.

## Comment

Solid pseudopapillary neoplasm is a rare tumour of the exocrine pancreas. It affects mostly young females between the second and third decade of life. SPNs are fundamentally solid tumours, although with increasing size, they become solid-cystic with internal haemorrhagic contents. The enhancement of the solid components is heterogeneous and typically hypovascular with progressive and heterogeneous fill-in. Most of the SPNs are surrounded by a well-defined enhancing capsule and frequently contains peripheral or central calcifications. Usually SPNs do not cause biliary or pancreatic duct dilatation or pancreatic atrophy. SPN can be localized in any portion of the pancreas.

Recently all SPN subtypes were classified as low-grade malignancies with excellent long-term clinical outcome [1]. Nevertheless, a limited number of SPNs may demonstrate aggressive pattern on histology, without any reliable distinctive feature on CT or MRI.

Although SPN is usually found in non-Causian women, the gender and age of our patient was typical for SPN. In additon, the absence of invasion into surrounding tissue and distant metastases were also in favour of the diagnosis of SPN. However, the imaging features of SPN and exophytic gastric GIST are similar. The enhancement in arterial phase suggests diagnosis of GIST, as SPN tends to be more hypovascular, with progressive and heterogeneous enhancement on portal venous phase. Preoperative biopsy should be considered in patients in whom the diagnosis is uncertain as it may affect the operative approach.
